# IGF-I regulates the age-dependent signaling peptide humanin

**DOI:** 10.1111/acel.12243

**Published:** 2014-07-18

**Authors:** Changhan Lee, Junxiang Wan, Brian Miyazaki, Yimin Fang, Jaime Guevara-Aguirre, Kelvin Yen, Valter Longo, Andrzej Bartke, Pinchas Cohen

**Affiliations:** 1 Leonard Davis School of Gerontology, University of Southern CaliforniaLos Angeles, CA, 90089, USA; 2 Children’s Hospital Los AngelesLos Angeles, CA, 90027, USA; 3 Geriatrics Laboratory, Department of Internal Medicine, Southern Illinois University School of MedicineSpringfield, IL, 62794, USA; 4 Universidad San Francisco de Quito, Avenida Vía Láctea en CumbayáQuito, Ecuador; 5 Instituto de Endocrinología IEMYRSan Ignacio, Quito, Ecuador

**Keywords:** aging, growth hormone, humanin, IGF-I, longevity

## Abstract

Aging is influenced by endocrine pathways including the growth hormone/insulin-like growth factor-1 (GH/IGF) axis. Mitochondrial function has also been linked to the aging process, but the relevant mitochondrial signals mediating the effects of mitochondria are poorly understood. Humanin is a novel signaling peptide that acts as a potent regulator of cellular stress responses and protects from a variety of *in vitro* and *in vivo* toxic and metabolic insults. The circulating levels of humanin decline with age in mice and humans. Here, we demonstrate a negative correlation between the activity of the GH-IGF axis and the levels of humanin, as well as a positive correlation between humanin and lifespan in mouse models with altered GH/IGF-I axis. Long-lived, GH-deficient Ames mice displayed elevated humanin levels, while short-lived GH-transgenic mice have reduced humanin levels. Furthermore, treatment with GH or IGF-I reduced circulating humanin levels in both mice and human subjects. Our results indicate that GH and IGF are potent regulators of humanin levels and that humanin levels correlate with lifespan in mice. This suggests that humanin represents a circulating mitochondrial signal that participates in modulating the aging process, adding a coordinated mitochondrial element to the endocrine regulation of aging.

## Introduction

The growth hormone and insulin-like growth factor-1 (GH/IGF-I) axis is a well-conserved endocrine system that regulates organismal size as well as lifespan (Fontana *et al*., 2010). Mutations in genes that regulate this endocrine signaling pathway have been shown to extend the lifespan of various model organisms. Emerging evidence suggests that certain mitochondrial factors could also add to the endocrine regulation of aging and aging-related diseases. Signals sent from the mitochondria to the cell are largely categorized as mitochondrial retrograde signals and have been thought to consist of nuclear-encoded proteins that reside in mitochondria, secondary and transient metabolites, and fragments of damaged mitochondrial DNA (mtDNA). Notably, in the nematode *C. elegans*, a stress-induced tissue-specific signal originating from mitochondria has been shown to communicate with distant organs and extend lifespan; the exact identity of this signal, termed ‘mitokine’, is unknown and currently being investigated (Durieux *et al*., 2011).

Humanin (HN) is the first reported retrograde signaling peptide that is highly likely to be encoded within the mtDNA and is found both in tissues and circulation (plasma) of rodents and humans (Hashimoto *et al*., 2001; Lee *et al*., 2013). The small open reading frame (smORF) sequence of HN fully identifies to the mtDNA. However, the possibility that the HN smORF originated from the mtDNA and transferred to the nuclear genome is not completely ruled out yet because of the existence of various nuclear DNA sequences of mitochondrial origin, a phenomenon known as NUMT; there are degenerate HN-like smORFs in the nuclear genome(Lee *et al*., 2013). The protective effects of HN have been faithfully replicated in laboratory settings both *in vitro* and *in vivo* (Lee *et al*., 2013). Interestingly, circulating levels of HN decline with age in mice and humans, indicating age-dependent regulation of its expression (Muzumdar *et al*., 2009). We set out to explore the possible interconnections of the GH pathway and the humanin system in the context of aging.

**Figure 1:**
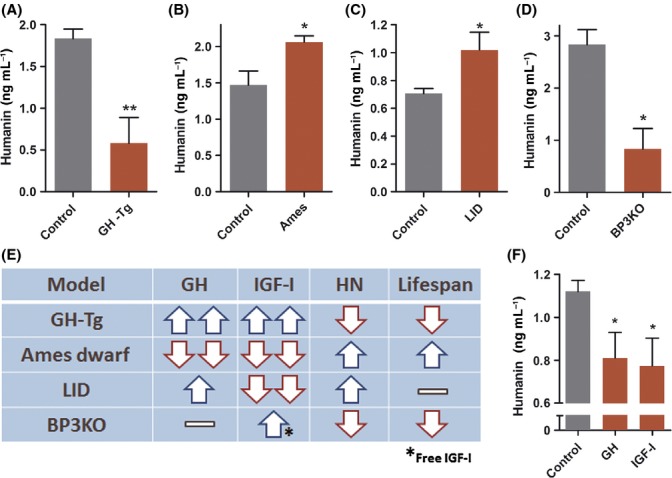
Humanin is positively correlated with lifespan and is regulated by the GH/IGF-I axis in mice. (A–D) Plasma humanin levels were measured in (A) GH transgenic mice (GH-Tg) (*N* = 11/group), (B) Ames dwarf mice (*N* = 10/group), (C) liver *igf1* gene deletion (LID) (*N* = 7/group), and (D) IGFBP-3 knockout (BP3KO) mice (*N* = 3/group); and respective matched controls.(E) Comparison of GH, IGF-I, HN levels and lifespan of each mouse model shown in panels A-D. (F) Male 12-week-old C57BL/6 mice (*N* = 8/group) were injected with human GH (2 mg kg^−1^ day^−1^, IP) or IGF-I (500 μg kg^−1^ day^−1^, BID, IP) for 20 days, and plasma was collected to measure humanin. Data are shown as mean ± SEM. Student’s *t*-test, *P < 0.05, **P < 0.01.

We studied the correlation between HN and lifespan using various mouse models with mutations in the GH/IGF-I axis, as these are among the most well-characterized genetic models of longevity. GH-transgenic mice (GH-Tg) have elevated levels of circulating GH and IGF-I, increased body size, experience premature and/or accelerated aging, higher tumor frequency, and have a ~50% reduced mean lifespan (Bartke, 2003). GH-Tg mice had a 70% reduction in plasma HN levels (Fig. 1A). Ames mice carry a homozygous mutation in the Prop 1 gene (Prop 1^df^) and as a result have nearly undetectable levels of GH and IGF-I, ~1/3 body weight, reduced tumor incidence, show delayed signs of aging, and have extended lifespan compared to normal controls (Bartke & Brown-Borg, 2004). Ames dwarf mice had a 40% increase in plasma HN levels (Fig. 1B). HN levels were negatively correlated with GH and IGF-I and positively correlated with lifespan in these models. Mice with liver *igf1* gene deletion (LID) have elevated levels of GH and an 80% reduction in IGF-I levels, and unexpectedly exhibit normal growth profiles and lifespan (Yakar *et al*., 1999). LID mice had a 45% increase in plasma HN levels (Fig. 1C). IGF binding protein 3 knockout mice (BP3KO) is another interesting model that has normal GH levels but enhanced IGF-I activity due to increased levels of free IGF-I, increased body size, and a reduced lifespan (Yakar *et al*., 2009; Mehta *et al*., 2011). BP3KO mice had a 70% decrease in plasma HN levels (Fig. 1D). The unique GH and IGF-I profile in each of these mouse models and their corresponding HN levels suggest IGF-I and not GH *per se* as the regulator of HN expression (Fig. 1E). To directly test the effect of GH and IGF-I on HN expression, we treated male C57BL/6 mice with rhGH (2 mg kg^−1^ day^−1^, IP) or rhIGF-I (500 μg kg^−1^ day^−1^, BID, IP) for 20 days. As suggested by the genetic models above, both GH and IGF-I treatment reduced plasma HN levels by ~30% (Fig. 1F) further suggesting that GH inhibits humanin via IGF-I.

**Figure 2:**
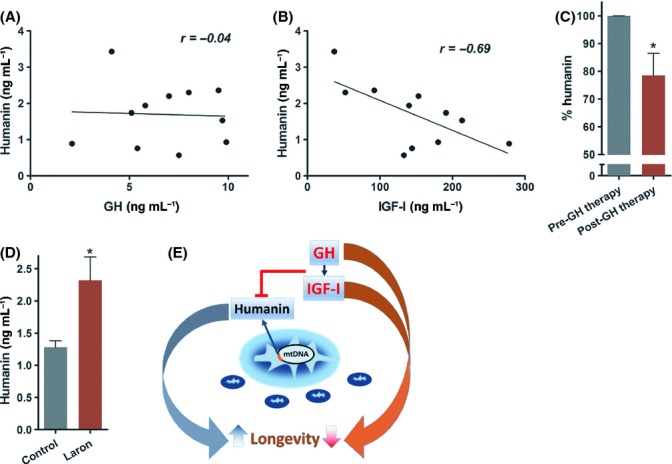
Humanin expression is regulated by the GH/IGF-I axis in humans. (A–C) Plasma humanin levels were measured from GH-deficient children (age 11.7 ± 1.8) before and after a 30-day GH treatment (*N* = 11/group). Pretreatment humanin levels were (A) not correlated with GH (*r* = −0.04, *P* = 0.9) but (B) highly negatively correlated with IGF-I (*r* = −0.69, *P* < 0.05). (C) Plasma humanin levels in these children were measured before and after GH treatment. (D) Plasma humanin levels of an Ecuadorian cohort with GH receptor deficiency (Laron Syndrome) and matched controls (*N* = 6/group). (E) Model of GH/IGF-I axis regulation of humanin and its effect on longevity. Data shown as mean ± SEM. Student’s *t*-test, *P < 0.05.

To extend our studies to humans, we collected plasma from untreated GH-deficient children who were being evaluated for their short stature (age 11.7 ± 1.8) and measured HN, peak stimulated GH, and IGF-I. We found HN levels were significantly highly negatively correlated to IGF-I levels (Pearson’s correlation coefficient of −0.69, *P* < 0.05) (Fig. 2A–B). Initiation of clinical treatment with daily GH injections in these children for 4-weeks at a dose of 50 μg kg^−1^ day^−1^ led to a significant (~20%) decrease in plasma HN levels by (Fig. 2C). We also measured plasma HN levels in an Ecuadorian cohort with GH receptor deficiency (GHRD) characterized by extreme short stature, very low levels of IGF-I, and a remarkable lack of cancers (Guevara-Aguirre *et al*., 2011). GHRD subjects had an 80% increase in plasma HN levels compared to normal matched Ecuadorian relatives (Fig. 2D).

We have previously shown that HN expression declines with age and have now demonstrated that genetic mutations in the GH/IGF-I axis that lead to extended lifespan or healthspan in mice and humans have elevated circulating HN levels compared to their wild-type counterparts. GH and IGF-I injections in mice corroborate the genetic models and suggest that HN is under direct IGF-I regulation. Therefore, it is interesting that HN and IGF-I levels concurrently decrease with age. This could be due to the age-dependent accumulation of mitochondrial DNA (mtDNA) damages including deletions and point mutations (Kennedy *et al*., 2013) as well as a decrease in mitochondrial number and mtDNA copy number in certain organs of rodents and humans (Bratic & Larsson, 2013). Notably, Ames dwarf mice exhibit much reduced levels of mtDNA damage and higher levels of circulating HN compared to their controls (Sanz *et al*., 2002).

It is also interesting to note that certain age-related diseases, such as atherosclerosis, are associated with a reduction of HN levels in human subjects, a fact that may contribute the mechanism by which it is associates with longer lifespan in mice (Oh *et al*., 2011).

The protection from the aging process that appears to be conferred by HN may be part of a general protective effect that HN has over various toxic insults, such as chemotherapy (Cohen, 2014).

Remarkably, HN has recently been shown to not just protect from the untoward effects of cancer chemotherapy, but to also reduce the growth of cancer xenografts in SCID mice (Eriksson *et al*., 2014), an observation that may explain the co-occurrence of reduced cancer and high HN levels in the Laron Syndrome cohort and Ames mice described here. It is also compelling to consider the preventive effects of HN in both Alzheimer’s disease models and diabetes models (Hashimoto *et al*., 2001; Muzumdar *et al*., 2009) as related to the longevity connection we demonstrate in this paper.

In summary, HN biology represents a novel mitochondrial paradigm that shifts our current view of mitochondria from a cellular energetics engine prone to wear-and-tear to an organelle with specific signaling peptides highly likely to be encoded within its own genome that are secreted and act as hormones (Fig. 2E). Future lifespan and healthspan studies on additional models such as HN transgenic or knockdown mice would be of great interest. We hypothesize that HN may be a novel mitochondrial candidate longevity gene.

## Experimental procedures

### Animals

12-week-old male C57BL/6 mice were purchased from Jackson Laboratories. All animal work was performed in accordance with the University of Southern California Institutional Animal Care and Use Committee and Animal Research Committee (ARC) protocols of the University of California, Los Angeles and according to NIH guidelines. Mice were intraperitoneally injected for 20 days with recombinant human IGF-I (rhIGF-I) at 500 μg kg^−1^ day^−1^ (BID) and recombinant human (rhGH) at 2 mg kg^−1^ day^−1^. Following 20 days of rhGH and rhIGF-I treatment, blood collected in K_2_-EDTA coated tubes (BD, Franklin Lakes, NJ, USA) by cardiac puncture at the time of euthanasia were immediately centrifuged for 10 min at 1500 g using a refrigerated centrifuge to obtain plasma. Plasma from 16-week-old male LID mice, 16-week-old female PEPCK-bGH mice, 24-week-old male Ames mice, 11-week-old male IGFBP-3 KO (BP3KO) mice, and their respective age- and sex-matched matched controls were collected using the same procedures. All genetically altered mice were of the C57BL/6 background.

### Human subjects

All studies have been reviewed and approved by the institutional IRB. Patients evaluated for short stature and GH-insufficiency were seen at the UCLA Pediatric Endocrinology clinic and enrolled after informed consent at the time of a growth hormone stimulation test. Plasma was collected before and 4 weeks after initiation of recombinant growth hormone at a dose of 50 mcg kg^−1^ days^−1^ according to routine clinical management.

### Immunoassays

Mouse and human IGF-1 and GH ELISA were measured by an in-house ELISA as previously described (Chin *et al*., 2013). Plasma HN levels were measured by an in-house HN ELISA using rabbit anti-HNG (HN analog) polyclonal antisera (Harlan Laboratories, Indianapolis, IN, USA). First, IgG-purified sera using a protein-A column (Pierce Chemical, Rockford, IL, USA) were used as capture antibodies. For the detection antibody, IgG-purified anti-HNG sera was further affinity purified using an HNG-conjugated ligand affinity column and subsequently labeled with biotin. Synthetic HN (Bachem, Torrance, CA, USA) was used to create a standard curve (0.1–50 ng mL^−1^). Plasma HN was extracted in 90% acetonitrile, and 10% 1 N HCl. Briefly, 200 μL of extraction reagent was added to 100 μL of plasma, gently mixed, and incubated at room temperature for 30 min. The mixture was centrifuged and the supernatant was removed and dried. The dried extract was reconstituted with 200 μL of phosphate buffer (50 mm sodium phosphate, 150 mm sodium chloride, 0.5% Tween-20, pH 7.6). 96-well microtiter plates were coated with capture antibody at 0.5 μg per well in 200 μL of 50 mm sodium bicarbonate buffer, pH 9.5, incubated 3–4 h at room temperature on a shaker, and then washed with wash buffer followed by 2 washes with Superblock buffer (Pierce Chemical Co). Standards, controls or extracted samples, and pretitered detection antibody were added to the appropriate wells and incubated overnight. Wells were then added with streptavidin-horseradish peroxidase conjugate after wash and further incubated for 30 min at room temperature. After 4 washes with wash buffer, 200 μL per well of o-phenylenediamine hydrochloride solution (1 mg mL^−1^ in hydrogen peroxide substrate) was incubated for 10–20 min. The reaction was terminated by the addition of 50 μL per well 2 N H_2_SO_4_, and absorbance was measured on a plate spectrophotometer (Molecular Designs) at 490 nm. It is worth noting that some variability observed in the humanin ELISA is largely because the data were collected over several years. While the plate-to-plate variability of the humanin assay is estimated to be <10% when compared at any particular point, the drift over time is greater. Importantly, all samples from each model were simultaneously measured within the same ELISA plate.

### Statistical analysis

Data are expressed as mean ± SEM. Data were analyzed using student’s *t*-test. Correlation analysis was done using Pearson correlation coefficient. A difference with *P*-values <0.05 were considered statistically significant. Statistical analyses were performed using GraphPad Prism version 5.0 software.
